# Relationship between the fasting status during hospitalisation, the length of hospital stay and the outcome

**DOI:** 10.1017/S0007114522000605

**Published:** 2022-12-28

**Authors:** Yuka Muto, Ayano Kurosawa, Chieri Ukita, Norio Hanafusa, Satoru Nagata

**Affiliations:** 1 Division of Clinical Nutrition, Tokyo Women’s Medical University, Shinjuku-ku, Tokyo, Japan; 2 Division of Clinical Nutrition, International University of Health and Welfare, Narita-shi, Chiba, Japan; 3 Department of Hemodialysis and Apheresis, Tokyo Women’s Medical University, Shinjuku-ku, Tokyo, Japan; 4 Department of Paediatrics, Tokyo Women’s Medical University, Shinjuku-ku, Tokyo, Japan

**Keywords:** Fasting period, Hospital stay, Home discharge rate, Mortality, Private university hospitals

## Abstract

The effects of long-term fasting on the prognosis and hospital economy of hospitalised patient have not been established. To clarify the effects of long-term fasting on the prognosis and hospital economy of hospitalised patients, we conducted a prospective observational study on the length of hospital stay of patients hospitalised at thrity-one private university hospitals in Japan. We conducted a prospective observational study on the effects of fasting period length on the length of hospital stay and outcome of patients hospitalised for 3 months in those hospitals. Of the 14 172 cases of hospitalised patients during the target period on the reference day, 770 cases (median 71 years old) were eligible to fast for the study. The length of hospital stay for fasting patients was 33 (4–387) days, which was about 2·4 times longer than the average length of hospital stay for all patients. A comparative study showed the length of hospital stay was significantly longer in the long-term-fasting (fasting period > 10 d; *n* 386) group than in the medium-term-fasting (< 10 d; *n* 384) group (median 21 v. 50; *P* < 0·0001). Although the discharge to home rate was significantly higher in the medium-term-fasting group (71·4 % *v*. 36·5 %; *P* < 0·0001), the mortality rate was significantly higher in the long-term fasting group (10·8 % *v*. 25·8 %; *P* < 0·0001). It was verified that the longer the fasting period during hospitalisation, the longer the length of hospital stay and lower home discharge rate, thus indicating that patient quality of life and hospital economy may be seriously dameged.

Several studies have reported that nutritional status during hospitalisation is closely related to treatment efficacy, length of stay and mortality^(1–4)^.

Although it is desirable to take meals orally, as a nutrition management method during hospitalisation, enteral nutrition and intravenous nutrition may be selected, depending on the disease or treatment method. As a rule, the intestinal tract is generally used when the intestinal tract is functioning, and it is considered clinically possible that intestinal mucosal homeostasis is maintained by the administration of nutrients into the intestinal tract, resulting in the maintenance of mechanical and immunological barrier functions^(5,6)^. Furthermore, it has been reported that starting enteral nutrition early reduces the incidence of infectious complications and improves the prognosis^(7–11)^. Conversely, there have been no clear clinical studies as of yet on the superiority of enteral nutrition over intravenous nutrition^(12–15)^.

Therefore, there is a tendency for the judgement of fasting or the period of fasting, along with nutritional management during the fasting period, to be different among facilities and among medical departments. To clarify the effects of long-term fasting on the prognosis and hospital economy of hospitalised patients, we investigated changes in BMI, haematological parameters, administered nutrients, components, etc., to examine the relationship between the inpatient fasting period, length of stay and outcome.

To demonstrate that proper nutritional management of inpatients leads to a better prognosis for patients, ultimately bringing financial benefits to hospitals, the effects of the duration of inpatient fasting on the length of hospital stay and outcome were investigated. The primary outcome was set to verify that the longer the fasting period, the longer the length of hospital stay, while the secondary outcome was to verify that the longer the fasting period, the lower the weight, the lower the haematological parameters and the lower the discharge to home rate.

## Materials and methods

### Ethics

This study was a multi-institutional joint study among private university hospitals in Japan and was implemented in accordance with World Medical Association’s Declaration of Helsinki upon receiving approval from TWMU Ethics Committee, Approval Number 4963-R2 followed by the ethics review committees of each institution. Informed consent was not obtained from each patient because as per the ethics committee of the university it was unnecessary but opt-out methods in this study.

### Target patients

The subjects consisted of patients who had fasted for at least ten consecutive meals, before and after dinner on the reference date (a specific day in July 2017), and patients aged 1 or older who had consumed three meals a day, among the inpatients at the study target facilities. Fasting was defined as the absence of using the gastrointestinal tract for oral intake, nasal nutrition, gastrostomy, etc.

### Target facilities

Of the eighty facilities registered with the Japan Private Medical Universities Nutrition Study Group, thirty one facilities (611 ± 385 approved hospital beds) cooperated in the study. The number of dietitians was 9·2 ± 7·9 per facility. 93 % of facilities had operating Nutrition Support Team (NST), while 56 % of facilities were determined to have added a NST, 72 % had ward charge systems for supervising dietitians and 14 % had registered dietitians stationed in the ward.

### Observation period

The observation period was set from the hospitalisation date to the observation end date (3 months after the reference date). For patients who continued hospitalisation after the observation end date, the observation end date was set as the date of clinical outcome and observation was discontinued. Outcomes were categorised into five types: hospitalised; discharged to home; discharged to a nursing home (discharge to facilities); transferred and died.

### Information collection method

At each target facility, the survey items of the study subjects were input and collected in unified Microsoft Office Excel 2013.

### Survey item

We investigated the target patients’ age, sex, medical department in charge (medical department), medical reasons for fasting (reasons for fasting), length of hospital stay, days from admission to the start of fasting, fasting days and the days from the end of fasting to the outcome. The nutritional administration status during the fasting period was investigated based on the nutritional administration method (no administration, peripheral venous nutrition, central venous nutrition (including use with peripheral venous nutrition)), energy administration per day, ratio of administered energy to basal metabolism (energy administration/basal metabolism), administered protein mass and use or non-use of fat preparations. Biochemical findings included BMI, serum Alb^(16,17)^ and peripheral blood Hb, at the time admission and outcome^(18–20)^.

### Statistical analysis

The results of the survey items were described as the mean (±standard deviation) for variables that were normally distributed among quantitative variables, and the median (minimum–maximum) for variables that were not normally distributed. Less than 5 % was considered statistically significant, in this test, using a two-group comparison with the Wilcoxon signed rank test, *t* test and X^2^ test. With respect to the survival analysis, a Cox proportional hazards model was used. Covariates were adjusted for age, sex, medical department, reason for fasting, BMI at admission, Alb at admission, Hb at admission, nutritional administration method, administered energy/basal metabolic rate and the use or non-use of fat preparations.

Propensity score matching was performed to match the patient background of fasting patients. The matching items were age, sex, BMI at admission, Alb at admission and Hb at admission. The overall treatment variables were applied to the patients who had fasted less than 10 d (hereinafter, medium-term fasting group) and patients who had fasted for 10 d or more (hereinafter, long-term fasting group). The effects on hospitalisation days and outcomes, depending on the fasting period, were analysed using the propensity score calculated from the logistic regression model. Survival time was analysed as a log rank test and sensitivity analysis, while death was defined as a competitive event, in this test, and analysed using Gray’s test. The Cochran–Mantel–Haenszel test was performed for stratified analysis in propensity score matching. JMP Pro 14.0.0 and SAS University Edition were used for the analysis.

## Results

### Overview of fasting patients

The number of patients who were provided meals on the reference day was 12 462, while the number of patients who were instructed to fast was 1710. 770 (5·4 %) of these patients had fasted for more than ten meals.

The median age was 71 years and included 474 men (61·6 %) and 296 women (38·4 %). The median length of stay among fasting patients was 33 d (4–387), approximately 2·4 times longer than the average length of stay of 13·9 d among all hospitalised patients.

### Outcomes of fasting patients

The discharge to home rate was 53·8 %, whereas the transfer rate was 19·8 %, the mortality rate was 18·4 % and 8 % continued hospitalisation on the end day of observation.

### Comparison of medium-term and long-term fasting groups

Because the median fasting period of fasting patients was 10 d, the medium-term (3–10 d fasting period: *n* 384) group was defined as the medium-term fasting group, while the long-term (fasting period 11–194 d: *n* 386) group was defined as the long-term fasting group for comparison. There was no significant difference between the two groups in terms of age and sex (Table 1).

**Table 1.** Comparison of fasting patient groups

Characteristics	Medium-term fasting group		Long-term fasting group		*P*
*n*	384		386		—
Age (years)	71	2–111	70	1–98	—
Sex (M:F)	233:151		241:145		—
Fasting days (days)	5·7	3·3–9·7	23·3	10–193·7	< 0·0001
Length of hospital stay (days)	21	4–184	50	12–387	< 0·0001
Hospitalisation–fasting start (days)	1	0–175	1	0–291	0·0006
Reason for fasting: Surgery-related (%)	34·4		12·7		< 0·0001
Gastrointestinal tract rest (gastrointestinal bleeding, etc.) (%)	31·5		35·0		—
End of fasting–outcome (days)	10	0–89	8	0–89	0·0006
BMI change rate (%: Hospitalisation–at the time of outcome)	–3·4	–31–21	–5·8	–34–23	0·0014
Serum Alb change rate (%: Hospitalisation–at the time of outcome)	–9·4	–65–118	–14·3	–76–113	0·0012
Blood Hb change rate (%: Hospitalisation–at the time of outcome)	–5·3	–64–159	–8·4	–52–159	0·0038
Amount of energy administration (kcal/d)	473	0–2017	623	0–1989	< 0·0001
Administered protein (g/d)	39	0–77	22·9	0–69	< 0·0001
Fat formulation usage (%)	5·5		33·2		< 0·0001
Total parenteral nutrition usage rate (%)	12·2		63·0		< 0·0001
Home discharge rate (%)	71·4		36·8		< 0·0001
Mortality (%)	10·9		25·8		< 0·0001

#### Fasting reason

The reason why ‘intestinal tract was unavailable’ was related to ‘surgery’ in 35 % of patients in the medium-term fasting group and 13 % in the long-term fasting group. The most common reason that surgery was not related was ‘gastrointestinal dysfunction due to the underlying disease,’ with 32 % in the medium-term fasting group and 35 % in the long-term fasting group.

#### Energy intake

Although the average daily energy administration was significantly higher in the long-term fasting group than in the medium-term fasting group (*P* < 0·0001), neither group reached basal metabolism. The average daily protein administration and the usage rate of fat preparations were significantly higher in the long-term fasting group than in the medium-term fasting group (*P* < 0·0001, respectively).

#### Nutrition methods

The use of central venous nutrition was significantly higher in the long-term fasting group than in the medium-term fasting group (12·2 % *v*. 62·9 %: *P* < 0·0001).

#### Nutrition evaluation

The long-term fasting group had significantly lower figures in terms of BMI, serum Alb value and peripheral blood Hb value (BMI: *P* = 0·0014, serum Alb: *P* = 0·0012), peripheral blood Hb: *P* = 0·0038), respectively, at the time of outcome than admission, compared with the medium-term fasting group.

#### Length of hospital stay

The length of hospital stay was significantly longer in the long-term fasting group than in the medium-term fasting group (*P* < 0·0001).

### Outcome

The discharge to home rate was significantly higher in the medium-term fasting group than in the long-term fasting group (71·4 % *v*. 36·5 %, (*P* < 0·0001)). Conversely, mortality was significantly higher in the long-term fasting group than in the medium-term fasting group (10·8 % *v*. 25·8 %, *P* < 0·0001).

The hazard ratio between the two groups was calculated to demonstrate that items other than fasting days (such as age and nutritional status at admission) did not significantly affect the probability of being discharged. The results indicated hazard ratio, 3·34; 95 % CI, 2·55, 4·38 (*P* < 0·0001) when covariates were considered, and hazard ratio, 3·82; 95 % CI, 2·94, 4·97 (*P* < 0·0001) after propensity score matching (Table 2).

**Table 2.** Cox comparison of probability of discharge using proportional hazard model

Parameters	Group	Number of people	Hazard ratio	95 % confidence interval	*P*
No adjustment	Medium-term fasting group	386	–	–	–
	Long-term fasting group	384	4·1	3·32, 5·03	< 0·0001
Covariate*	Medium-term fasting group	314	–	–	–
	Long-term fasting group	313	3·4	2·55, 4·38	< 0·0001
Propensity score matching**	Medium-term fasting group	231	–		–
	Long-term fasting group	231	3·8	2·94, 4·97	< 0·0001

* Covariates included age, sex, BMI at admission, Alb at admission, Hb at admission, presence or absence of fat preparations, nutritional administration method and energy dose/basal metabolism.

** The matching items were age, sex, BMI at admission, Alb at admission and Hb at admission.

**Fig. 1.**
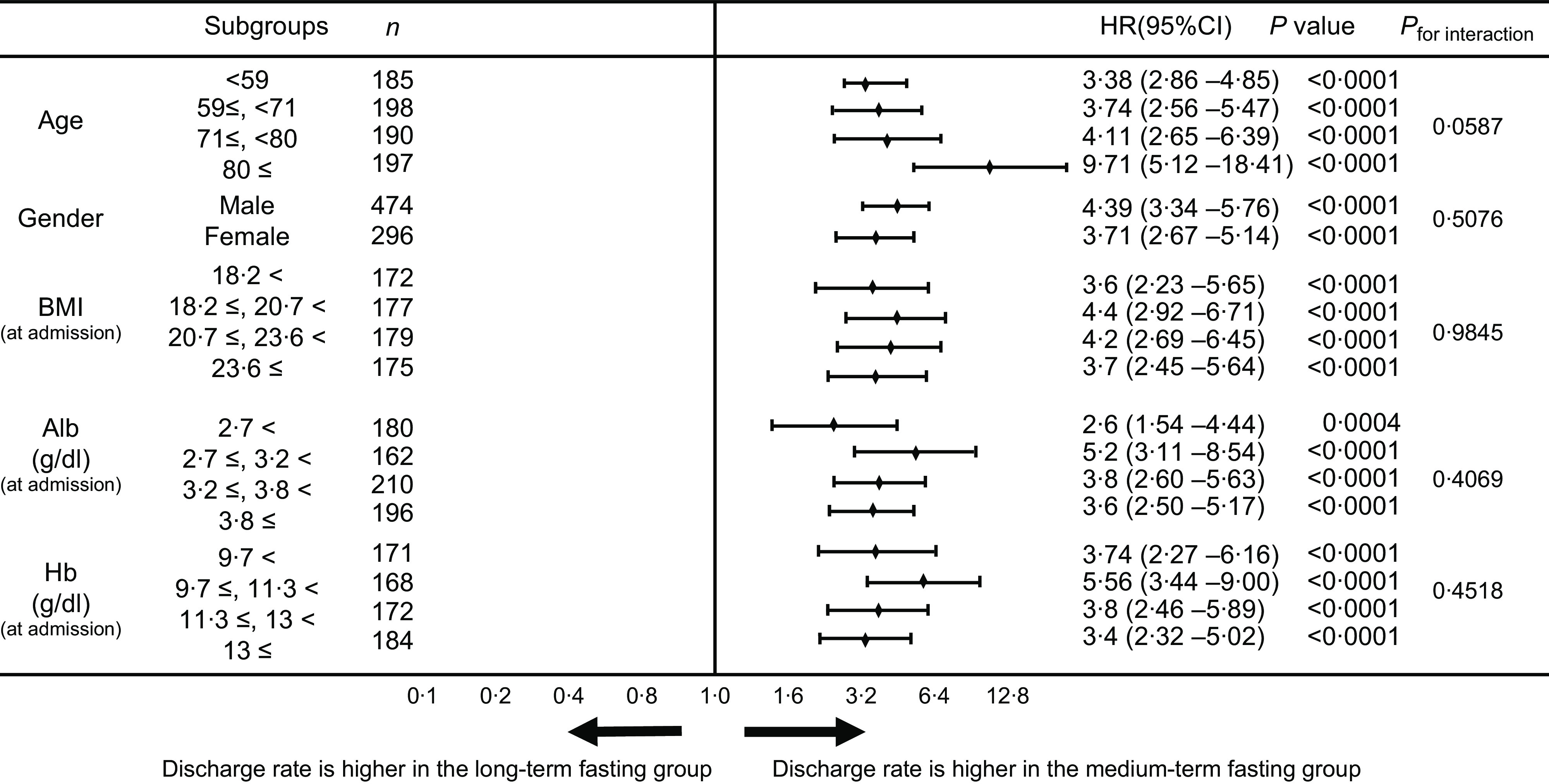
Comparison of probability of discharge from hospital using hazard ratios of BMI and haematological parameters.

**Fig. 2.**
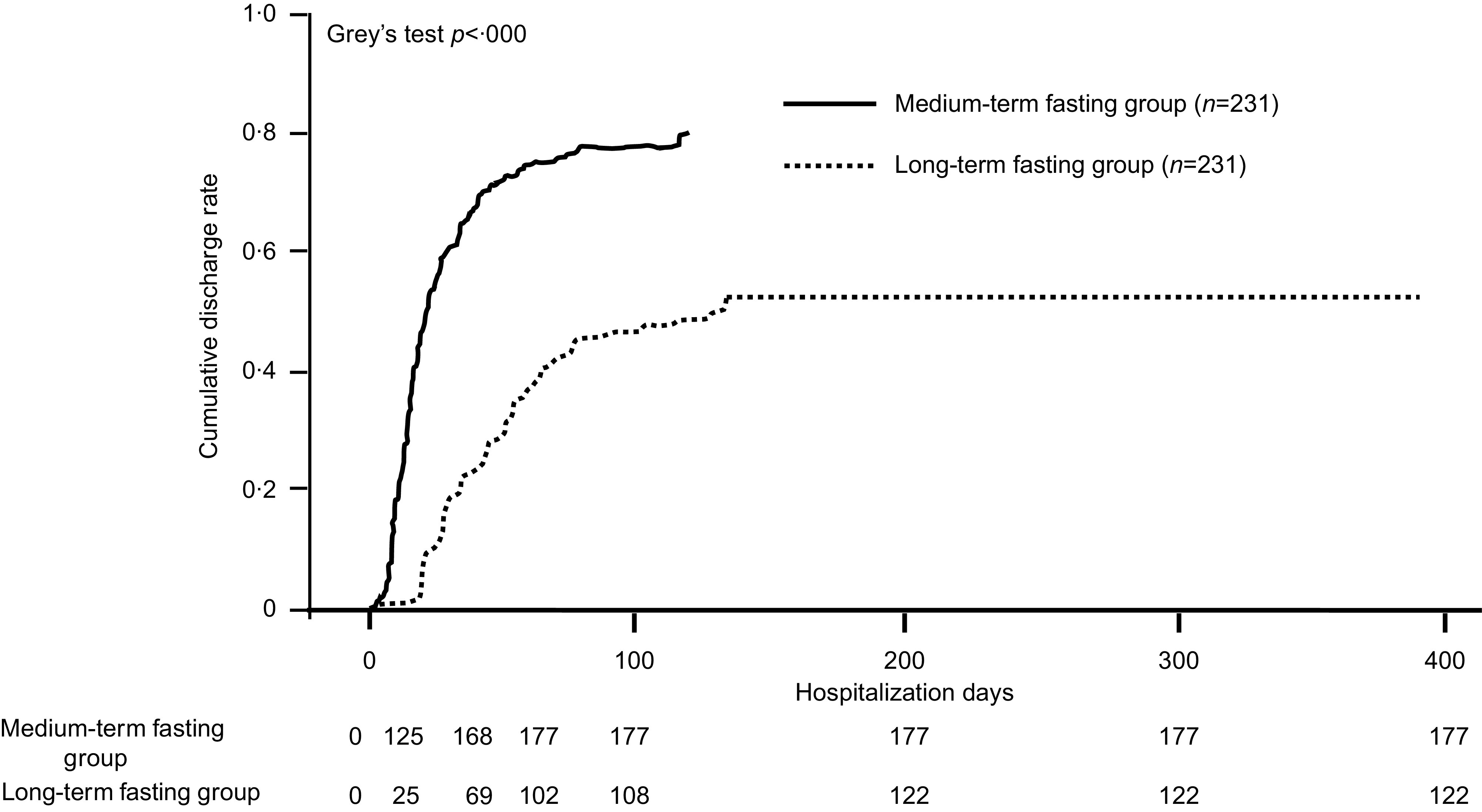
Survival time analysis for competing risk events.

The medium-term fasting group was associated with higher discharge rates than the long-term fasting group in all subgroups (Fig. 1). Furthermore, the survival time analysis at competitive risk events, using the data after propensity score matching, indicated that the cumulative discharge rate was significantly higher in the medium-term fasting group than in the long-term fasting group (Fig. 2; *P* < 0·0001)

As a result of the survival time analysis for competitive risk events, using the data after propensity score matching, the cumulative discharge rate was significantly higher in the medium-term fasting group than in the long-term fasting group.

## Discussion

In Japan, due to the benefits of the universal health insurance system, the burden on patients for hospitalization costs is low and the length of hospital stay tends to be longer than in other countries^(21)^. Moreover, the use of intravenous nutrition formulas in Japan is higher than in other countries because much of the expenses are covered by health insurance^(22)^.

However, prolonged use of intravenous nutrition can lead to the extinction of gastrointestinal function and immunity, resulting in reduced patient Quality of Life (QOL) and wasted hospital expenses due to bloodstream infections and complications^(23–27)^.

Because it is difficult to clarify the income and expenditure situation at each hospital, the calculation of ‘number of hospital stays’ can be cited as a powerful tool that reflects this.

Because private hospitals affiliated with private universities in Japan are of a certain size and tend to have similar business conditions, the length of hospital stay is generally constant, making it suitable for estimating the impact of certain conditions on the hospital economy. Here, the authors focus on patient discharge rates and hospital stays as a measure of the impact of patient fasting on QOL and hospital profits. To demonstrate that the fasting period can severely damage the nutrition of patients, we examined changes in BMI and blood parameters.

As a result, the length of hospital stay for fasting patients was more than 2·4 times longer than the average length of hospital stay at the targeted private university hospitals. To find out if the longer the fasting period, the longer the hospital stay, a comparison was made between the medium-term fasting group with a fasting period of less than 10 d and the long-term fasting group with more than 10 d, the results of which indicated that the length of hospital stay was significantly extended in the latter. Therefore, the possibility was suggested that the longer the fasting period, the lower the hospital’s profit, in Japan.

Next, we investigated whether the fasting period during hospitalization affects the discharge to home rate of patients and it was verified that prolonging the fasting period significantly reduces the patient discharge to home rates and furthermore increase mortality. Furthermore, it has been reported that intervention by a dietitian has contributed to the improvement of the nutritional status of patients^(28–30)^. Going forward, we would like to examine the relationship between the active intervention of dietitians, the length of stay among patients and the discharge to home rates.

Lastly, to clarify that the reason why longer fasting periods lead to longer lengths of hospital stays and clearly the deteriorated nutritional status of patients in hospital, we compared changes in BMI, serum Alb and peripheral blood Hb, that are haematological parameters, at the time of outcomes among medium-term and long-term fasting groups. As a result, it was revealed that the longer the fasting period, the significantly lower the BMI, serum Alb level, and peripheral blood Hb level at the time of outcome, compared with the time of admission, and that fasting clearly worsens the nutritional status of the patient. It was also highlighted that patients were discharged from hospital with poor nutrition. It should be noted here that intravenous energy administration, the amount of administered protein and the amount of fat were significantly higher in the long-term fasting group than in the medium-term fasting group. In other words, no matter how much the nutritionally correct amount of energy is calculated and components are contrived, there is little benefit to patients and hospitals in providing them intravenously. The reason for this is that nutrition through the gastrointestinal tract is most physiological in humans, helping to assure homeostasis of metabolism and immune activity, as verified by a variety of evidence. However, it is difficult to verify that this theory is correct, even in actual clinical settings, with this study being the first to prove it with a sufficient number of samples, as far as we know^(31)^.

As this study was all conducted by registered dietitians at private university hospitals in Japan, the authority to conduct the survey was limited. With respect to the haematological parameters, the ESPEN provides evidence for nutritional evaluation, the use of the CONUT score (consisting of serum Alb value, peripheral blood total lymphocyte counts and serum total cholesterol value), which provided evidence for nutritional evaluation with ESPEN, could have provided a simpler study with more evidence. However, the preliminary surveys indicated that routine measurements of total cholesterol levels at each facility were low and had to be excluded from this survey.

This study was associated with some limitations. First of all, since this study was a simple observational study, comparisons could not be carried out regarding various key items such as the length of hospital stay under the same background conditions, such as the severity of disease in the fasting and non-fasting groups. In addition, it was not possible to exclude the possibility that the fasting group may have been affected by a group with serious diseases compared to the non-fasting group. Going forward, we plan to collaborate with the NST of all hospitals to assess the severity of the fasting group and non-fasting group and to also investigate the length of hospital stay, BMI and CONUT score, using a prospective study design.

### Conclusion

As a result of the investigation on the effect of the duration of fasting by hospitalised patients, on the length of hospital stay and outcome, it has been verified that the longer the fasting period during hospitalisation, the longer the length of hospital stay, weight loss, decreased haematological parameters and lower home discharge rates.
